# Analysis of Fuel Cell Stack Performance Attenuation and Individual Cell Voltage Uniformity Based on the Durability Cycle Condition

**DOI:** 10.3390/polym13081199

**Published:** 2021-04-08

**Authors:** Chunjuan Shen, Sichuan Xu, Yuan Gao

**Affiliations:** 1 School of Automotive Studies, Tongji University, Shanghai 201804, China; cjshen@tongji.edu.cn (C.S.); scxu@tongji.edu.cn (S.X.); 2 Shanghai Ranrui New Energy Vehicle Technology Co., Ltd, Shanghai 201804, China; 3 Yangtze Delta Region Institute of Tsinghua University, Jiaxing 314006, China

**Keywords:** durability cycle, performance attenuation, voltage uniformity, variation coefficient

## Abstract

Based on the dynamic cycle condition test of a 4.5 kW fuel cell stack, the performance attenuation and individual cell voltage uniformity of the proton exchange membrane fuel cell (PEMFC) stack was evaluated synthetically. The performance decay period of the fuel cell stack was 180–600 h, the decrease of voltage and power was evaluated by rate and amplitude. The results show that the performance of the fuel cell stack decreased with the increase of test time and current density. When the test was carried out to 600 h, under rated operating conditions, the voltage attenuation rate was 130 μV/h, and the voltage reduced by 71 mV, with a decrease of 10.41%. The power attenuation rate was 0.8 W/h, with a decrease of 10.42%. The statistical parameter variation coefficient was used to characterize the voltage consistency of individual cells. It was found that the voltage uniformity is worse at the high current density point and with a long-running process. The variation coefficient was 3.1% in the worst performance.

## 1. Introduction

Fuel cell technology has been highly valued in recent years due to its high efficiency, low pollution, rapid and dynamic response, superior reliability and durability, demonstrating great advantages in space, automobiles, hydrogen-fueled motor vehicles and fuel cell hybrid vehicles [1]. But it is difficult for a single fuel cell to achieve the required voltage. In order to meet the working requirements, fuel cell stack composed of multiple single cells is often used to generate electricity or supply energy. Therefore, to make sure the fuel cell stack is in good condition, it is very important to measure the durability performance of both single fuel cell and fuel cell stack.

To evaluate the durability of proton exchange membrane (PEM) fuel cells, two types of methods are usually used: steady-state tests and accelerated stress tests (AST) [2]. These two test methods are suitable for two working modes of fuel cell vehicle separately. When the fuel cell vehicle is working at power follow mode, steady-state tests will be suitable. When the fuel cell vehicle is working at soft run mode, the fuel cell stack will provide a dynamic power, thus the ASTs is more suitable for this mode. In view of these two different situations, researchers have carried out many experiments to explore the influence of these factors on the attenuation of the whole fuel cell system by changing the humidity, temperature, external working environment and other conditions.

In order to test the steady-state attenuation lay of fuel cells, researchers have carried out many steady-state tests. The department of energy has announced the long-term durability target of >500 h for automobile applications, and more than 20,000 h for external environment conditions [3,4,5]. Bose et al. [6] and S.J. Cleghorn et al. [7] have conducted the durability test on a proton exchange membrane fuel cell (PEMFC), and the performance degradation rate of the cell was also evaluated. Pahon et al. [8] have carried out two micro-cogeneration (μ-CHP) durability tests, the voltage variation of fuel cell in 1000 h and 500 h under steady-state condition and the influence of long-term test duration shortening on voltage attenuation are analyzed. Cheng et al. [9] studied the fuel cell stack performance by examining the cell polarization curves under different tests lengths of time. Wahdame et al. [10] have carried out a durability test of fuel cell stack for 1000 hours at the test temperature of 55 °C and current density of 0.5 A/cm², the performance attenuation of fuel cell stack was studied Hou et al. [11] compared the performance degradation of fuel cell engine for passenger cars under the actual road conditions and bench test conditions. It is found that the performance attenuation rate under the actual road condition is about four times of that under the bench test condition at the current of 120 A. Liu.Y.N et al. [12] studied the law of performance attenuation under constant load of fuel cell system in a fixed power station. The voltage attenuation rate of the fuel cell stack was found to be 0.042 V/h and power attenuation rate of 2.8 W/h respectively. Jian Zhao et al. [13] studied the effect of wet-dry cycles through water intrusion-evaporation processes and water flow-through-dehydration experiments, respectively. The cycling of water intrusion-evaporation processes was found to contribute to the growth of agglomerates and the water flow-through-dehydration was found to enlarges the large pores but had less effect on agglomerate sizes. P Pei et al. [14] proposed a quick evaluating method for automotive fuel cell vehicles with an arithmetic equation of fuel cell lifetime. J Xie et al. [15] conducted test under high humidity conditions. Major factors of failure such as membrane degradation, dissolution of catalyst-layer recast ionomer, catalyst oxidation were also included. Chang et al. [16] proposed a mathematical model to evaluate the effects of clamping force, temperature and relative humidity on mechanical changes in the microstructure of catalyst layers, thus simulating the working situation under startup and shut down cycle.

Apart from the steady-state tests, accelerated stress test is also conducted to test the attenuation under dynamic condition. Yuan et al. [17] reviewed established conditioning protocols and reported methods to control PEM single cells and stacks to provide for accelerated conditioning techniques, so that tests can complete the process in a short time period. SJ Bae et al. [18] provided a straightforward accelerated degradation testing (ADT) procedure for PEMFC. The ADT procedure included statistical modeling of attenuation in PEMFCs under startup-shutdown cycling conditions. K Panha et al. [19] focused on accelerated durability tests with different modes of membrane failure via relative humidity cycling. I Bloom et al. [20] conducted tests using four duty cycles and the tests lasted 200 h for each duty cycle. The change in stack performance was also evaluated. Pei et al. [21] focused on the main factors affecting the life time of fuel cells on vehicles. The problems on water management and gas transport were studied in load dynamic cycling, which simulated the working progress of fuel cell vehicles. Lai et al. [22] studied the specific effects of isolated chemical and mechanical degradation stressors on the morphology and physical-chemical properties of fuel cell membranes. Two isolated accelerated stress tests were designed to test their effects. Y Jeon et al. [23] identified the new accelerated life-time (ALT) test protocols for polymer electrolyte membrane fuel cell. Degradation phenomena under different load step cycling conditions were studied. P Li et al. [24] conducted an extensive program of experimental and simulation work to find out the accelerated degradation in the fuel cell stacks. M Hicks et al. [25] conducted accelerated tests and built statistical lifetime model to evaluate durability of membrane electrode assemblies (MEAs).

In this paper, the fuel cell stack durability cycle test is carried out for 600 h. Based on the durability test data of fuel cell platform, the performance of fuel cell stack is analyzed, and the relevant parameters are evaluated with the corresponding indicators.

## 2. The Durability Cycle Test Setup

**Figure 1:**
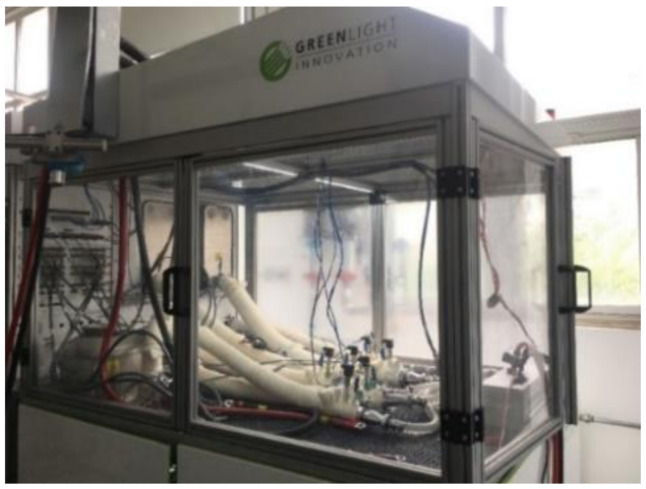
The test platform of durability cycle.

The platform for the durability cycle test of the fuel cell stack is FCATSTMG500, which is produced by the Greenlight Innovation Corp. (Burnaby, BC, Canada) which is shown in Figure 1.

The test bench is composed of gas supply system, cooling system, load system, control system, data acquisition system, safety system and many other modules, which can be used to perform the performance characterization test and airtight test of fuel cell stacks of 2~30 kW. The setting conditions of bench parameters are shown in Table 1.

In this paper, the fuel cell stack composed of 25 single-battery series is 488 mm long, 155 mm wide and 145 mm high. The rated power of the fuel cell stack is 4.5kW, and the effective working area is 312 cm^2^. The technical characteristics of the stack are shown in Table 2.

**Figure 2:**
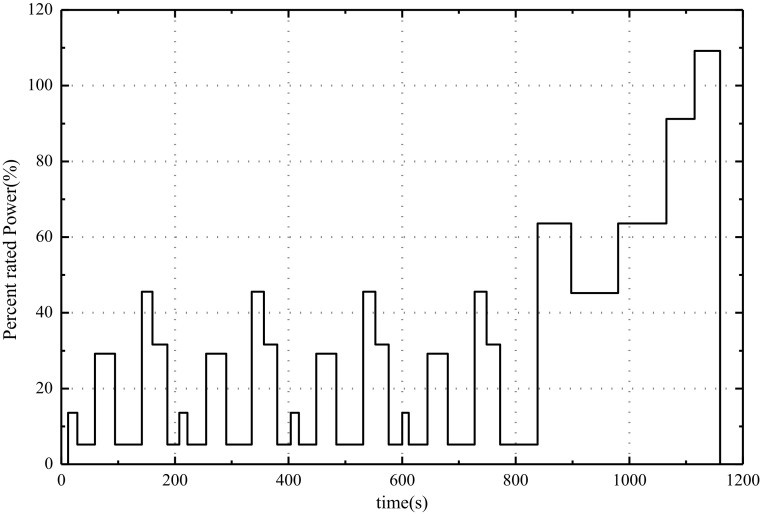
The durability test cycle of fuel cell stack.

In the fuel cell stack, deionized water is used as cooling water and composite material is used as bipolar plate. The operating conditions used in the fuel cell stack durability test are derived from the NEDC (New European Driving Cycle). In this paper, according to the characteristics of NEDC operating conditions and the actual vehicle driving conditions, the cycle condition of the vehicle fuel cell stack durability platform test is established, including idle speed, acceleration, deceleration, constant speed, the rated working conditions and other daily vehicle employment conditions [8]. The test working condition is shown in Figure 2.

As shown in Figure 1, the durability test loading conditions include idle operation, partial load condition, rated operating condition and overload condition, with a cycle time of 1200 s.

In the characterization technology of a fuel cell, the current-voltage response of the fuel cell, i.e. the polarization curve, reflects the overall performance of the fuel cell. In this paper, the cycle durability test of fuel cell stack has been carried out for 600 h, and 1800 cycles have been completed. In the first 630 cycles (0–210 h) of fuel cell operation, the polarization curve test is carried out per 90 cycles. After 630 cycles (210–600 h), the polarization curve test is carried out per 60 cycles. Therefore, 28 polarization curves are obtained.

## 3. Polarization Curve of Fuel Cell Stack

The polarization curves of each measurement are obtained by fitting the experimental data by the model of the polarization curve semiempirical models [9]. The semi-empirical model of the polarization curve is shown in the following Formula (1):

(1) $$E_{\mathrm{cell}}{=E}_{\mathrm{OCV}}-\mathrm{blog}\left(\left({i+i}_{\mathrm{loss}}\right)/i\right)-\mathrm{Ri}-m\left[\exp\left(\mathrm{ni}\right)-1\right]$$

where E_cell_ means fuel cell voltage; E_ocv_ means fuel cell open circuit voltage; i means current density; b means Tafel slope; i_loss_ means loss current density; R means cell resistance; m and n are constant, and represent concentration loss together.

**Figure 3:**
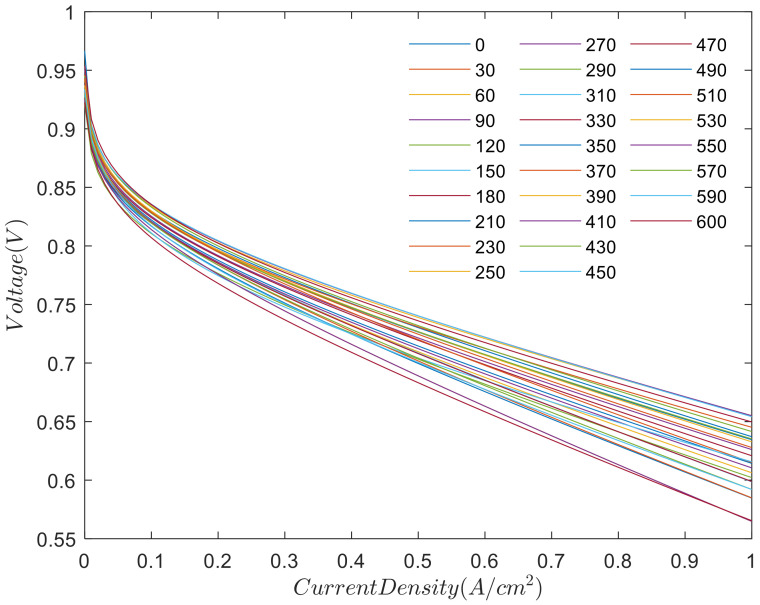
The polarization curve for durability test at different time stages.

According to polarization curve test data and the semiempirical model, the polarization curves are shown in Figure 3. We use the polarization curve (the conventional method of steady-state performance analysis) to analyze the electrical properties in the characteristics of the fuel cell reactor. We separate the analysis intervals into three parts: the polarization curve test is done every 30 h between 0 and 210 h, and conducted every 20 h between 210 and 600 h. Each polarization curves are shown in Figure 3.

As shown in Figure 3, the polarization curve of the fuel cell stack shows a downward trend, indicating that the performance of fuel cell stack becomes worse with the increase of test time.

## 4. Analysis and Evaluation of Fuel Cell Stack Performance 

### 4.1. Common Current Density

**Figure 4:**
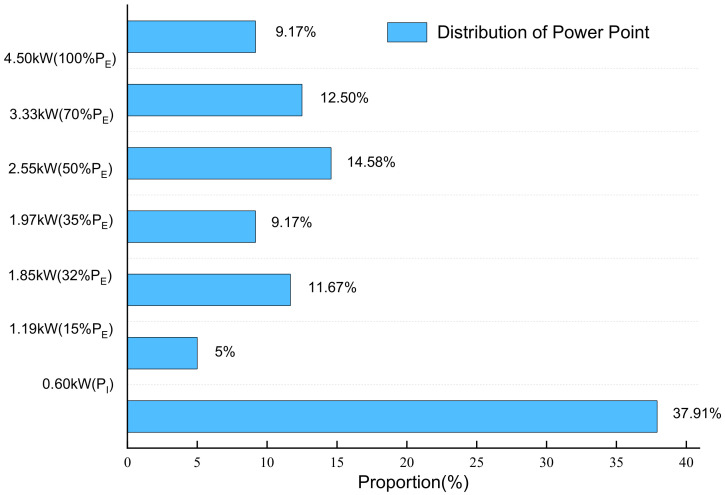
The distribution of load power.

According to the durability test loading conditions in Section 2, the power distribution can be obtained. The distribution of load power is shown in Figure 4.

As shown in Figure 4, the power of fuel cell stack durability cycle test is composed of the idle point P_I_, 15% P_E_, 32% P_E_, 35% P_E_, 50% P_E_, 70% P_E_ and rated Power P_E_. The current density corresponding to different power is taken as the common current density point of fuel cell stack durability test. Simultaneously, the open circuit state (the current density is 0) and the peak current density should also be the common current density.

In the durability cycle test, we calculated the current and voltage corresponding to each power to determine all common current density. The characteristic current density is shown in Table 3.

As shown in Table 3, the current density commonly used in fuel cell stack durability cycle test is determined. In addition to the common current density involved in cycle conditions, the current density corresponding to open-circuit voltage and peak power is added for a more comprehensive follow-up analysis.

### 4.2. Voltage Analysis of Fuel Cell Stack

**Figure 5:**
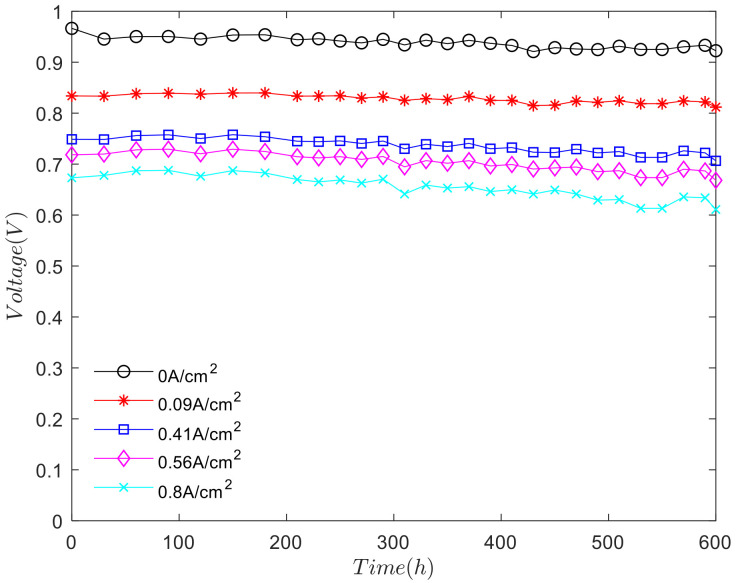
The variation of voltage over time under different current density.

The character current density was obtained from the working condition curve of the durability bench test of fuel cell stack. We analyzed the change of voltage with cycle time under characteristic current density, as shown in Figure 5.

As shown in Figure 5, except the open circuit voltage, the change trend of voltage at other current density points is consistent. During the first 180 hours, the voltage is rising. At this stage, the performance of the fuel cell stack rises slightly in the activation process. The activation process has an important influence on improving the performance of the fuel cell stack and which is beneficial to the performance of the fuel cell electrode. Between 180 and 450 h, the overall voltage of fuel cell stack presents the downward trend. The performance of the fuel cell stack begins to decline, but the decay process is slow. When the test was carried out to 450 and 600 h, the voltage of the fuel cell stack decreased obviously. The higher the current density, the more severe the voltage attenuation. Under the condition of the long-term durability cycle test, the fuel cell stack will produce the degradation of the electrode material, the rupture of the proton exchange membrane, which will increase the ohmic internal resistance and ohmic loss. At this time, the fuel cell stack is in the nonactivation period, also known as the performance degradation period.

#### 4.2.1. Voltage Attenuation under Fixed Currents

**Figure 6:**
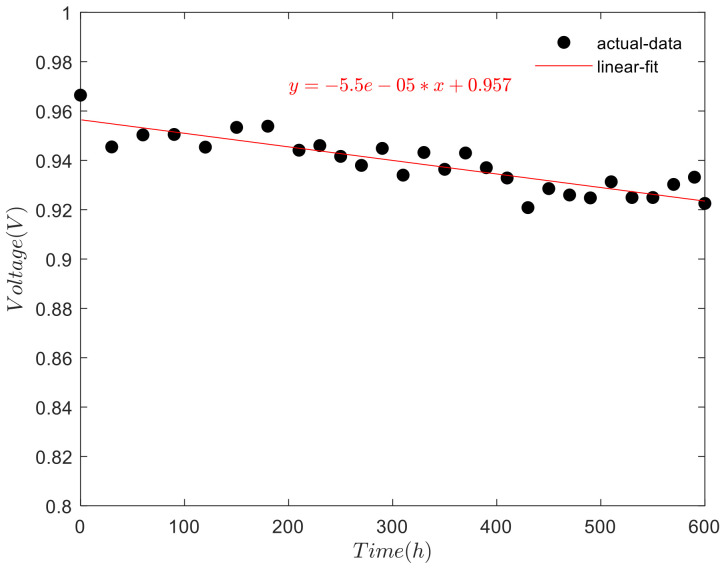
Open-circuit voltage changes over time.

When the fuel cell stack is in the open circuit state, the current density is 0, the voltage attenuation is different from other current density. The attenuation of open circuit voltage over test time is shown in Figure 6.

As shown in Figure 6, the open-voltage of the fuel cell stack s with the increase of the test time. The linear fitting of the open-circuit voltage shows a downward trend with the increase of test time. When the test is carried out to 600 h, under the open-circuit condition, the voltage of fuel cell stack decreased 43.8 mV. The voltage attenuation rate is 55 μV/h, with an attenuation amplitude of 4.5%.

The nonactivation period of 180–600 h was selected to study the performance degradation of the fuel cell stack, except the open circuit state of fuel cell. The voltage variation was characterized by the voltage attenuation rate and the voltage attenuation amplitude.

**Figure 7:**
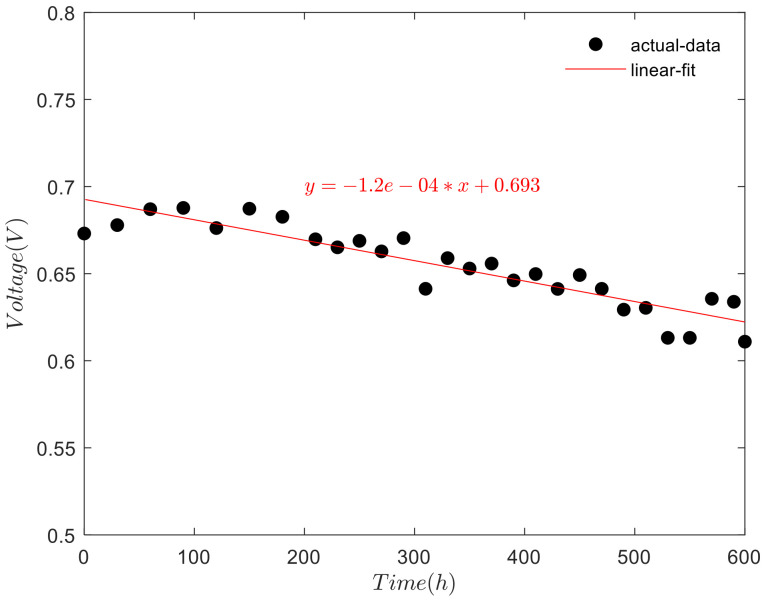
Idle voltage changes over time.

When the fuel cell stack operates at idle power, the current density is 0.09 A/cm^2^. At this time, the fuel cell stack has performance attenuation during the nonactivated period. The attenuation of idle voltage over test time is shown in Figure 7.

As shown in Figure 7, the linear fitting of the idle voltage shows a downward trend with the increase of test time in the nonactivation period. The experimental data are processed according to SSE (sum of square error):

(2) $$\mathrm{SSE}\;=\;\sum {\left(y_{exp}-y_{sim}\right)}^{2}$$

Under the same sample collection, the smaller the SSE value is, the better the fitting between the equation and the experimental results is. The value of SSE is 0.0023 < 0.05, with small error and good fitting effect:

(3) $$R^{2}\;=\;1-\;\frac{\sum {\left(y_{exp}-y_{sim}\right)}^{2}}{\sum {\left(y_{exp}-y_{ave}\right)}^{2}}$$

For R-square (Coefficient of determination). The closer the value of R is to 1, the stronger the interpretation ability of the fitting equation to the experimental results is. The results show that the R value is 0.9150 > 0.9, and the fitting equation has a strong ability to explain the experimental data. When the test is carried out to 600 h, the voltage of fuel cell stack decreased 28 mV. The voltage attenuation rate is 46 μV/h, with an attenuation amplitude of 3.34%.

**Figure 8:**
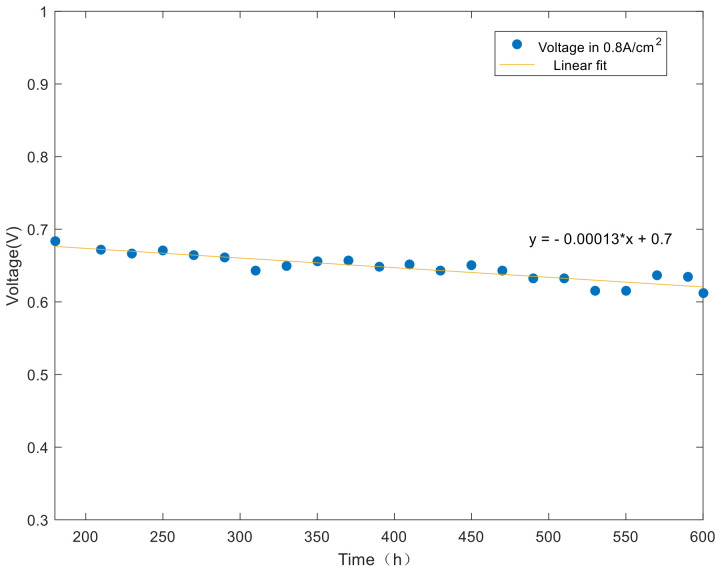
Rated voltage changes over time.

When the fuel cell stack operates at rated power, the current density is 0.8 A/cm^2^. At this time, the fuel cell stack has performance attenuation during the nonactivated period. The attenuation of rated voltage over test time is shown in Figure 8.

As shown in Figure 8, the linear fitting of the rated voltage shows a downward trend with the increase of test time in the nonactivation period. Similarly, the first test is based on the SSE formula. The result shows that the SSE value is 0.0013 < 0.0023 < 0.05, and the fitting result is better than the previous model, which is in line with the expectation. In addition, the equation’s R-square value is 0.9032 > 0.9. The fitting equation also has a strong ability to explain the experimental data. When the test is carried out to 600 h, the voltage of fuel cell stack decreased 71 mV. The voltage attenuation rate is 130 μV/h, with an attenuation amplitude of 10.41%.

#### 4.2.2. Whole Voltage Attenuation

**Figure 9:**
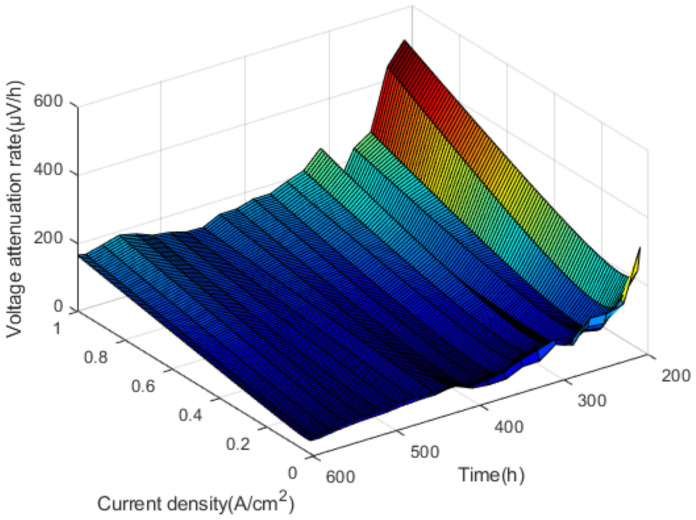
The voltage attenuation rates.

Within 180–600 h, the voltage decreases over time are in fluctuation, the voltage downward trend can be linear fitted, and the fitting line slope is the voltage attenuation rate. The voltage attenuation rates are shown in Figure 9.

As shown in Figure 9, by fitting the voltage attenuation rate, it is found that the change rate of voltage in different time periods shows a quadratic function change trend, that is, the increase of voltage attenuation rate accelerates with the increase of current density. In addition to the open-circuit voltage, the voltage attenuation rate corresponding to other current density reaches a maximum of 310 h in the test.

**Figure 10:**
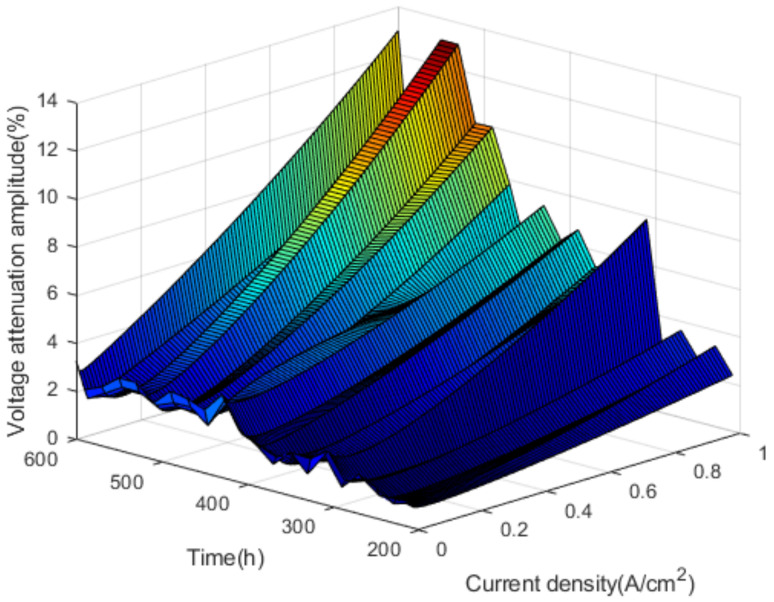
The voltage attenuation amplitudes.

In the nonactivation period, by calculating the voltage attenuation amplitude at any time within 180–600 h, the voltage attenuation amplitude corresponding to different time and current density is obtained. The voltage attenuation amplitudes are shown in Figure 10.

As shown in Figure 10, in the nonactivation period, the voltage attenuation amplitude fluctuates with the increase of current density and test time, and the overall trend is upward. In addition to the open-circuit voltage, the voltage attenuation amplitude corresponding to other current density reaches a maximum of 310 h in the test. At this time, the performance of the fuel cell stack begins to deteriorate significantly.

Based on the analysis of the voltage attenuation under the commonly used current density, it is found that the voltage of the fuel cell stack decreases with the increase of the test time. The degree, rate and amplitude of the voltage drop increase with the current density. At the same time, it is found that the voltage decay rate and amplitude can reflect the voltage change of the fuel cell stack, which can be used as the performance evaluation index of the fuel cell stack.

### 4.3. Power Analysis of Fuel Cell Stack

**Figure 11:**
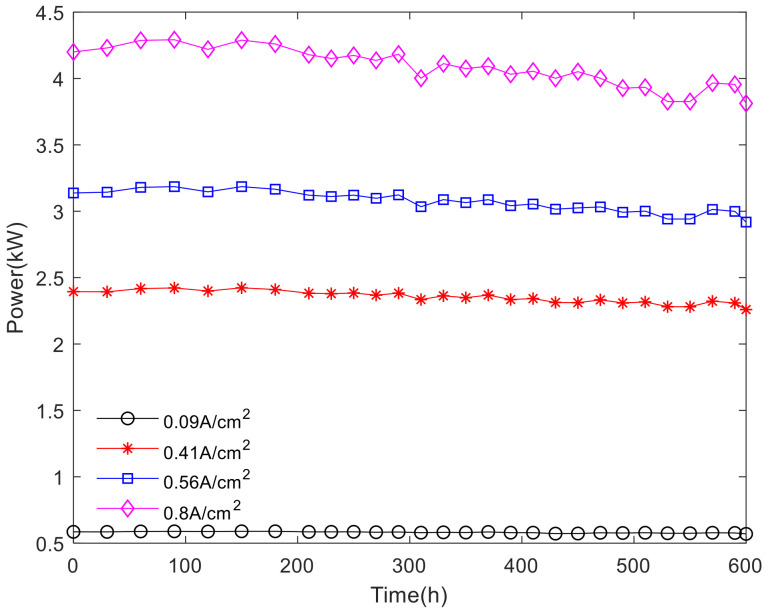
The power at different current density.

According to the polarization curve fitting of the fuel cell stack, the voltage and current of the fuel cell stack under different cycles were obtained, and then the power of the fuel cell stack was calculated. Power can reflect the output capacity of the fuel cell stack, the power output at the commonly used current density is shown in Figure 11.

**Figure 12:**
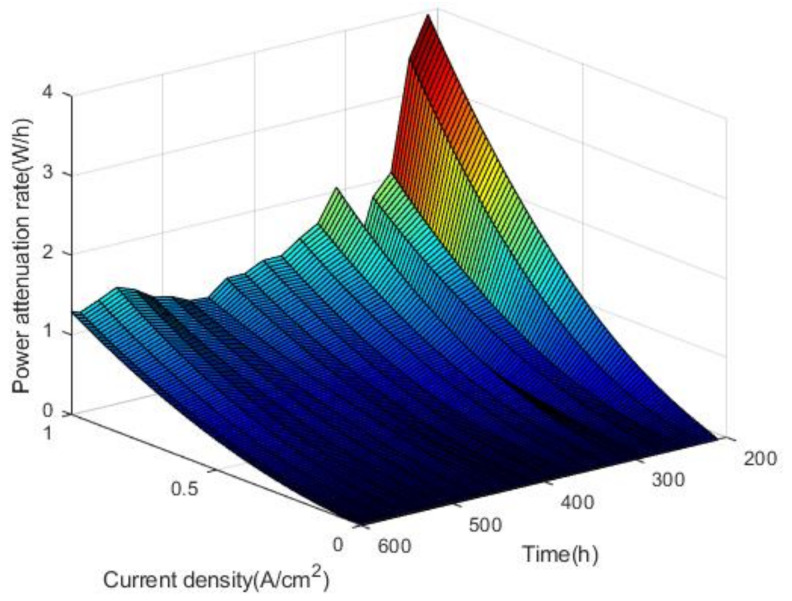
The power attenuation rates.

**Figure 13:**
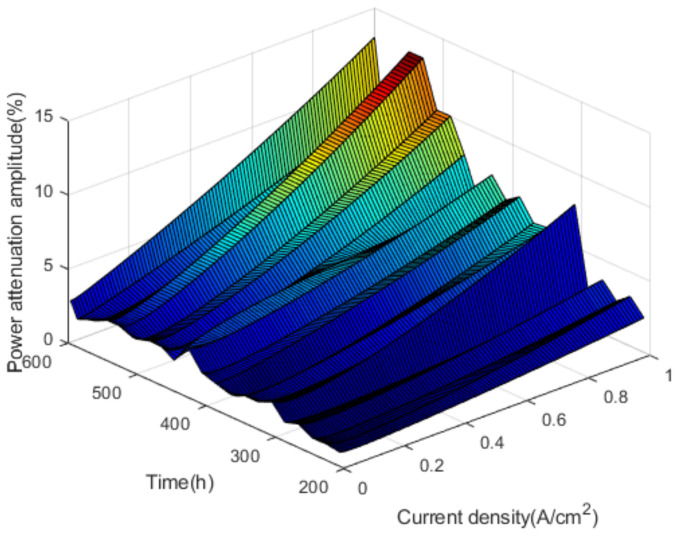
The power attenuation amplitudes.

As shown in Figure 11, power varies little with the increase of current density at medium and small current density. At high current density, there is a slight upward trend in the increase of power over time in the activation interval. Within the nonactivation interval, the power fluctuates with time, and the higher the current density is, the more obvious the power decline trend is. According to the calculation method of voltage attenuation rate and attenuation amplitude, the power attenuation rate and attenuation amplitude of the fuel cell stack can be obtained. The power attenuation rates and attenuation amplitudes are shown in Figure 12 and Figure 13.

As shown in Figure 12 and Figure 13, it can be concluded that in the nonactivated interval 180–600 h, the power has a tendency to decay, and the power attenuation rate increases with the current density. By fitting the power attenuation rate, it is found that the change conforms to the secondary function trend. At rated power, the power attenuation rate of the fuel cell stack reached 0.8 W/h and the power attenuation amplitude reached 10.42%.

## 5. Analysis of Individual Cell Voltage Uniformity of Fuel Cell Stack 

In statistics, statistical parameters can be used to analyze the voltage uniformity of fuel cell stack. Standard deviation reflects the degree of dispersion between individuals. In the performance analysis of the fuel cell stack, the coefficient of variation can reflect the degree of variation of different single-cell voltages. The variation coefficient is the relative standard deviation of a single battery’s voltage, and the advantage of the coefficient of variation over the standard deviation is that the average value of the sample is not required. When a stack which consists of N chips of fuel cell works under a certain current density, the voltage of each single cell forms a sample, variation coefficient of the sample is shown in the following formula.

(4) $$C_{V}=\frac{S}{\bar{x}}\times 100\%=\frac{\frac{\sqrt{\sum_{i=1}^{N}{\left(x_{i}-\bar{x}\right)}^{2}}}{N-1}}{\bar{x}}\times 100\%$$

where S means standard deviation; $\bar{x}$ means average single-cell voltage of fuel cell stack in sample; N means number of single cells; *x*_i_ means single-cell voltage.

Analysis of fuel cell stack single-chip voltage uniformity in nonactivated intervals, where a single battery voltage drop affects the performance of the fuel cell stack. At current density of 0, 0.09 A/cm^2^, 0.18 A/cm^2^, 0.8 A/cm^2^, the variation coefficient of the single battery in the fuel cell stack is shown in Figure 13.

**Figure 14:**
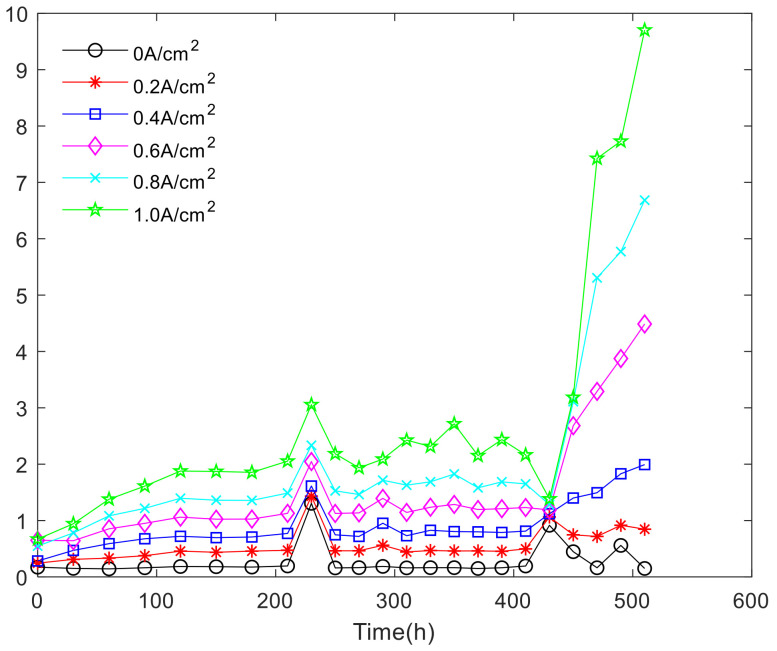
The coefficient of variation change over time.

**Figure 15:**
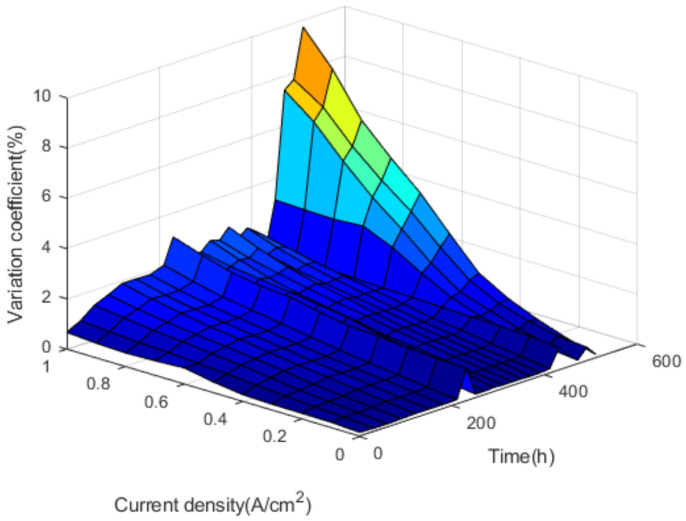
The overall variation coefficient.

As shown in Figure 14, it can be discovered that the coefficient of variation increases with the increase of current density in 180, 210, 250, 290, 330, 370, 410, 450, 530, 600 h, that is, the uniformity of fuel cell stack is worse with the increase of current density. The results show that the voltage uniformity of fuel cell stack becomes very poor under high current density. Figure 15 shows the overall variation coefficient of the fuel cell stack cell with time and current density.

As shown in Figure 15, the coefficient of variation rises slowly at the beginning and sharply at the end of the test run. When the cycle condition goes on some time, the coefficient of variation suddenly rises and then drops, which means there are many single cell voltage changes in fuel cell stack. At this time, the voltage uniformity of a single cell is unstable, which will lead to the performance deterioration of the fuel cell stack. Under the condition of high current density, the increase of variation coefficient is very obvious. This is mainly due to the uneven gas reaction within the fuel cell stack in the high current density. The complex and unstable transmission process of generated water quality makes the performance difference of each single cell larger.

## 6. Conclusions

In this study, the data of the fuel cell stack durability cycle test were analyzed. The current density in common use under durability cycle condition was found out, and the attenuation rate and attenuation amplitude of different parameters were used to evaluate the performance attenuation of fuel cell stack. The coefficient of variation was used to evaluate the single voltage uniformity of the fuel cell stack.

According to the voltage change slot under the common current density, it was found that in 0–180 h, the fuel cell stack is in the activation period and the voltage is on the rise. In the 180–600 h period, the fuel cell stack is in the nonreactive phase, the voltage is decreasing and the performance is beginning to decay. During the performance attenuation period, the decrease of voltage and power of the fuel cell stack is characterized by the attenuation rate and the attenuation amplitude. The voltage and power attenuation rate in different time periods show the trend of the secondary function with the increase of current density, which means the attenuation rate of the indicator shows the trend of accelerating and rising with the increase of current density. At the time of 300 h, the attenuation rate of each index reaches the maximum. At 600 h, the attenuation amplitude of each index reaches the maximum.

We selected the daily work point for analysis, that is, we obtained the total fuel cell reactor voltage under different current density points. At the same time, it was found that when the fuel cell reactor outputs power externally, the performance increases between 0 and 180 h, and, between 180 and 600 h performance decreases. Preliminary analysis of the fuel cell reactor after the factory needs to go through the active period (0–180 h), the active period for the fuel cell reactor to run to the best function of the required time period. During this period, the fuel cell reactor physical property, machinery and other performance are the best, got the highest external output power. After 180 h, the voltage is reduced due to the attenuation of materials.

Through this durability cycle test, it was found that the greater the coefficient of variation, the worse the voltage uniformity of the fuel cell stack. When in medium and large current density, the voltage uniformity of the fuel cell stack decreases slowly over at the beginning of its life, and there is no big change in the medium period. At the end of its life, there is a drastic decline of the voltage uniformity over time.

## Figures and Tables

**Table 1:** Operating conditions of the proton exchange membrane fuel cell (PEMFC) stack test.

Parameters	Values
Relative humidity of hydrogen	without humidification
Relative humidity of gas	80%
Stoichiometry of hydrogen	1.2
Stoichiometry of air	2.5
Inlet temperature of hydrogen	60 °C
Inlet temperature of air	60 °C
Inlet pressure of hydrogen	50 kPa
Inlet pressure of air	without pressurization
Outlet temperature of coolant	60 °C

**Table 2:** Technical characteristics of stack.

Physical characteristics	Length	357 mm
	Width	490 mm
	Height	180 mm
	Number of fuel cells	75
	Effective working area of membrane(cm^2^)	**-**
Electrochemical characteristics	Rated power	6.55 kW
	Rated current	135 A
	Rated voltage	48.53 V
	Rated efficiency	51.76%
	Peak power	7.35 kW
	Peak current	160 A
Operating conditions	Fuel	Mixture of hydrogen and nitrogen or reforming product, target hydrogen volume ratio: 80–100% (dry)
	Operating pressure	<300 mbar (g)
	Relative humidity	80–100% RH
	Oxidant	Air
	Operating pressure	<300 mbar (g)
	Maximum pressure difference between hydrogen and oxygen	200 mbar (g)
	Relative humidity	95–100% RH
	Coolant	Deionized water and/or propylene glycol or ethylene or ethylene glycol, <50% ethylene glycol
	Fuel cell temperature	50–60 °C (inlet)

**Table 3:** The power, voltage and current of the PEMFC stack.

Name	Characteristic Power	Power (kW)	Average Single Cell Voltage (V)	Current Density (A/cm^2^)	Current (A)
Open circuit voltage	0	0	0.96	0.	0.0
Idle speed	Idle Power	0.6	0.82	0.09	28.9
Common power	15% P_E_	1.19	0.79	0.18	56.5
	32% P_E_	1.85	0.77	0.29	90.2
	35% P_E_	1.97	0.76	0.31	96.0
	50% P_E_	2.55	0.74	0.41	128.0
	70% P_E_	3.33	0.71	0.56	173.2
Rated power	P_E_	4.5	0.66	0.80	249.6
High Power	Peak power	4.9	0.63	1.00	312.0

## Data Availability

The data presented in this study are available on request from the corresponding author.
